# The efficacy and safety of glucokinase activators for the treatment of type-2 diabetes mellitus: A meta-analysis

**DOI:** 10.1097/MD.0000000000027476

**Published:** 2021-10-08

**Authors:** Qian Gao, Wenjun Zhang, Tingting Li, Guojun Yang, Wei Zhu, Naijun Chen, Huawei Jin

**Affiliations:** Affiliated Hospital of Shaoxing University of Edocrine and Metabolism Department, Zhejiang, China.

**Keywords:** glucokinase activators, meta-analysis, type-2 diabetes mellitus

## Abstract

**Background:**

Glucokinase activators (GKAs) are a novel family of glucose-lowering agents used for the treatment of type-2 diabetes mellitus. Treatment with different GKAs has been shown to reduce blood glucose levels in these patients. We compared the efficacy/safety of GKAs in patients with type-2 diabetes mellitus through a meta-analysis.

**Methods:**

We searched the PubMed, Excerpt Medica Database, and Cochrane Central Register of Controlled Trials databases for articles published before December 30, 2020. We computed the weighted mean difference (WMD) and 95% confidence interval (CI) for the change from baseline to the study endpoint for GKA versus placebo treatments.

**Results:**

A total of 4 articles (5 studies) were included in the meta-analysis. GKAs were associated with reductions in glycated hemoglobin levels from baseline (WMD, −0.3%; 95% CI, −0.466% to −0.134%). No significant difference between GKA and placebo treatment was observed in the results of fasting plasma glucose levels from baseline (WMD 0.013 mmol/L; 95% CI, −0.304–0.33 mmol/L). A significantly higher change in 2-hour postprandial plasma glucose (2-h PPG) levels (WMD −2.434 mmol/L; 95% CI, −3.304 to −1.564 mmol/L) was observed following GKA than placebo treatment. GKAs were associated with a higher prevalence of causing hypoglycemic events than placebo treatment (risk difference [RD], 0.06; 95% CI 0.013–0.106). GKAs had no association with the risk of developing adverse effects (RD, 0.038; 95% CI, −0.03–0.106) and serious adverse events (RD, 0.01; 95% CI, −0.004–0.023).

**Conclusions:**

GKAs were more effective for postprandial blood glucose control. However, these agents showed a significantly high risk of causing hypoglycemia.

**PROSPERO registration number:**

CRD42021220364.

## 1 Introduction

Glucokinase activity is associated with glucose-regulated insulin and glucagon secretion in the pancreas, with the enzyme acting as a glucose sensor. In the liver, glucokinase processes glucose after a meal and converts it into glycogen by acting as a glucose sensor in pancreatic β-cells. Therefore, glucokinase has a central role in glucose homoeostasis, with the blood glucose levels being maintained at 4 to 6.5 mmol/L.^[1]^ The mutation of the human glucokinase gene causes a decrease in glucokinase activity in β-cells and thus an increase in the threshold of blood glucose, which leads to moderate fasting hyperglycemia in young patients with maturity-onset diabetes.^[2]^ It has been clinically demonstrated that impaired glucokinase function can cause glucose metabolic diseases, including type 2 diabetes mellitus (T2DM).^[3–5]^ Glucokinase activators (GKAs) can effectively reduce glycosylated hemoglobin (HbA_1c_) levels and improve β-cell function in patients with T2DM by improving the function of glucokinase. The low affinity of GKAs to glucose makes them show significant activity only at high glucose concentrations, indicating that these molecules may have a low risk of causing hypoglycemia. However, in some clinical trials, the incidences of hypoglycemia following treatment with piragliatin and MK-0941, novel GKAs, were high.^[6,7]^ At the same time, in some long-term administration studies, it was found that 2 GKAs, AZD1656 and MK-0941, lost efficacy after several months of therapy.^[7,8]^ However, the dorzagliatin study of Hualing medicine, which completed a phase III clinical trial of the molecule, showed that HbA_1c_ levels can decrease by up to 1.12% after oral administration of the GKA at a dose of twice daily.^[9,10]^ In addition, an increase in the dosage showed an increase in the proportion of patients whose HbA_1_c levels dropped to within the standard range, while the incidence of hypoglycemia did not significantly increase. In order to provide new medical evidence for the management of T2DM, we undertook a meta-analysis to assess the efficacy and safety of GKAs in patients with the disease.

## 2 Methods

### 2.1 Study registration

This systematic review and meta-analysis protocol was registered in the International Prospective Register of Systematic Reviews database (registration no. CRD42021220364; https://www.crd.york.ac.uk/prospero/#recordDetails).

### 2.2 Search strategy

We conducted a search of the PubMed, Excerpt Medica Database, and Cochrane Central Register of Controlled Trials databases for articles published before December 30, 2020 using the following search terms: “glucokinase activator,” “Dorzagliatin,” “HMS5552,” “Piragliatin,” “RO4389620,” “AMG 151,” “ARRY-403,” “AZD1656,” “AZD6370,” “TMG-123,” “MK-0941,” “TTP-399,” “SY004,” “GKM001” for randomized controlled trials (RCTs) in patients with T2DM. The detailed search strategy is shown in Table 1.

**Table 1:** Search strategy used in this study.

Literature databases	Search items
PubMed	(“Diabetes Mellitus, Type 2”[MeSH Terms] OR “Type 2 Diabetes Mellitus” OR “NIDDM” OR “Type 2 Diabetes”) AND (“glucokinase activator”[MeSH Terms] OR “Dorzagliatin” OR “HMS5552” OR “Piragliatin” OR “RO4389620” OR “AMG 151” OR “ ARRY-403” OR “AZD1656” OR “AZD6370” OR “TMG-123” OR “MK-0941” OR “TTP-399” OR “SY004” OR “ GKM001”) AND clinical trial[ptyp]
EMBASE	(“Diabetes Mellitus, Type 2”/exp OR “Type 2 Diabetes Mellitus” OR “NIDDM” OR “Type 2 Diabetes”) AND (“glucokinase activator”/exp OR “ Dorzagliatin”:ti,ab,kw OR “ HMS5552”:ti,ab,kw OR “ Piragliatin”:ti,ab,kw OR “RO4389620”:ti,ab,kw OR “ AMG 151”:ti,ab,kw OR “ARRY-403”:ti,ab,kw OR “ AZD1656”:ti,ab,kw OR “ AZD6370”:ti,ab,kw OR “ TMG-123”:ti,ab,kw OR “MK-0941”:ti,ab,kw OR “ TTP-399”:ti,ab,kw OR “SY004”:ti,ab,kw OR “ GKM001”:ti,ab,kw) AND ’randomized controlled trial’/de
CENTRAL	((Diabetes Mellitus, Type 2):ti,ab,kw OR (Type 2 Diabetes Mellitus):ti,ab,kw OR (NIDDM):ti,ab,kw OR (Type 2 Diabetes):ti,ab,kw) AND ((glucokinase activator):ti,ab,kw OR (Dorzagliatin):ti,ab,kw OR (HMS5552):ti,ab,kw OR (Piragliatin):ti,ab,kw OR (RO4389620):ti,ab,kw OR (AMG 151):ti,ab,kw OR (ARRY-403):ti,ab,kw OR (AZD1656):ti,ab,kw OR (AZD6370):ti,ab,kw OR (TMG-123):ti,ab,kw OR (MK-0941):ti,ab,kw OR (TTP-399):ti,ab,kw OR (SY004):ti,ab,kw) OR (GKM001):ti,ab,kw

CENTRAL = Cochrane Central Register of Controlled Trials, EMBASE = Excerpt Medica Database.

### 2.3 Study selection

Studies were eligible for inclusion if they: were RCTs; compared a GKA with a placebo; treated patients for ≥12 weeks; had at least 1 baseline and post-treatment efficacy and/or safety outcome of interest; included patients with T2DM aged ≥18 years; were published in English.

### 2.4 Data extraction

Two independent reviewers extracted the following information from articles that met the inclusion criteria: publication information (first author's name and year of publication); baseline characteristics of the study (study size, ages of the participants, duration of follow-up, and country); outcomes regarding efficacy and safety (change from baseline to the study endpoint for levels of HbA_1c_, fasting plasma glucose, 2-hour postprandial plasma glucose [2-h PPG], hypoglycemic events, adverse events [AEs], serious adverse events [SAE]).

If the same trial reported data at different follow-up durations, we extracted the data corresponding to the longest follow-up period. If a study reported results on the effects of GKAs at different doses, we combined subgroups for analysis of each dose.

### 2.5 Quality assessment

Articles meeting the inclusion criteria were assessed using the 5 items of scale proposed by Jadad for published studies that evaluate randomization (0–2 points), double-blinding (0–2 points), and description of withdrawals (1 point).^[11]^ Scores ranging 0 to 5 and a score ≥3 indicated that a study was of “high quality.” The score was not used as a criterion for study selection; it was used only for descriptive purposes. Disagreements between the reviewers were discussed until a consensus was reached.

### 2.6 Statistical analyses

Continuous data were summarized as the weighted mean difference (WMD) with 95% confidence intervals (CIs) for the change from baseline to the study endpoint for GKAs versus placebos. Dichotomous data were summarized as the risk difference (RD) with a 95% CI. If the 95% CI included a value of 0, we considered the difference between the GKA and placebo not to be significant. Heterogeneity was assessed using the *Q*-statistic and *I*^2^ metric (*I*^2^ values of 25%, 50%, and 75% were considered to indicate “low,” “medium,” and “high” heterogeneity, respectively) among trials. A *P-*value of the *Q*-statistic < 0.1 and an *I*^2^ > 50% represented “substantial variability” and a random-effect model was used, otherwise, a fixed-effect model was applied.^[12,13]^ If heterogeneity was >50%, we performed subgroup analyses. To estimate a possible publication bias caused by the tendency of published studies to be positive, Egger and Begg tests were used and the level of HbA_1c_ was considered to be the main variable outcome.^[14]^ Sensitivity analyses were undertaken by omitting one study at a time and computing the pooled effect size of the remaining studies to evaluate if the results were markedly affected by a single study. All analyses were done using Stata v11.0 (Stata, College Station, TX). This meta-analysis was conducted according to the Preferred Reporting Items for Systematic Reviews and Meta-analyses (PRISMA) statement.^[15]^

## 3 Results

### 3.1 Characteristics of enrolled studies

**Figure 1:**
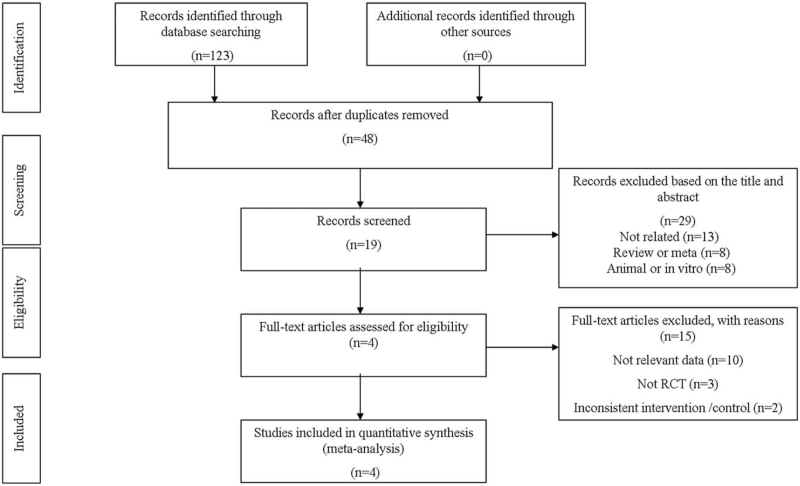
Flowchart of the included studies.

A total of 123 potential articles were extracted and evaluated. Following this, 4 articles (5 studies) presenting RCTs were included in our meta-analysis.^[6–8,10]^ The search results are summarized in Fig. 1 and the baseline characteristics of included studies are described in Table 2. Of these 5 studies, the investigated GKAs were PF-04937319 (N = 2), MK-0941 (N = 1), AZD1656 (N = 1), and dorzagliatin (N = 1).

**Table 2:** Characteristics of the RCTs included in the meta-analysis.

Author	Participants	Mean age	Mean HbA1c	Mean ± SD FBS	Mean ± SD 2h-PPG	Int	Follow-up		Jadad
year	(Int/Ctrl,N)	(Int/Ctrl, years)	(Int/Ctrl, %)	(Int/Ctrl, mmol/L)	(Int/Ctrl, mmol/L)	Ctrl	(weeks)	Country	score
Amin NB	160/56	56/55	8.0/7.9	9.4 ± 2.3/9.0 ± 1.7	–	PF-04937319	12	America, Canada	4
2005–1^[15]^						Placebo			
Amin NB	200/46	48/48	8.1/8.1	9.1 ± 2.5/9.3 ± 2.4	–	PF-04937319	12	America, Canada	4
2005–1^[15]^						Placebo			
GE GEMM	472/115	56/56	8.6/8.6	8.5 ± 2.4/8.4 ± 2.5	15.4 ± 3.5/15.1 ± 3.3	MK-0941	14	27 countries	4
2001^[17]^						Placebo			
Wilding JP	307/47	59/57	8.3/8.4	9.0 ± 2.3/8.9 ± 2.2	–	AZD1656	16	Europe, Latin America	4
2013^[18]^						Placebo			
Zhu D	202/53	56/55	8.4/8.5	9.6 ± 2/9.3 ± 1.8	17.6 ± 3.2/17.0 ± 3.7	Dorzagliatin	12	China	5
2018^[19]^						Placebo			

2-h PPG = 2-hour postprandial plasma glucose, HbA1c = glycated hemoglobin, RCT = randomized controlled trial.

Table 2 shows the major characteristics of the included studies. All 5 studies were RCTs. The basic information included first author, country, study duration, number of participants at baseline, age, and intervention information. The duration of these studies varied from 12 to 30 weeks.

### 3.2 Methodological quality

According to the evaluation of the methodological quality of the included RCTs, all studies scored >3 points and were considered to be of “high quality.”

### 3.3 Efficacy outcomes

#### 3.3.1 HbA_1_c

**Figure 2:**
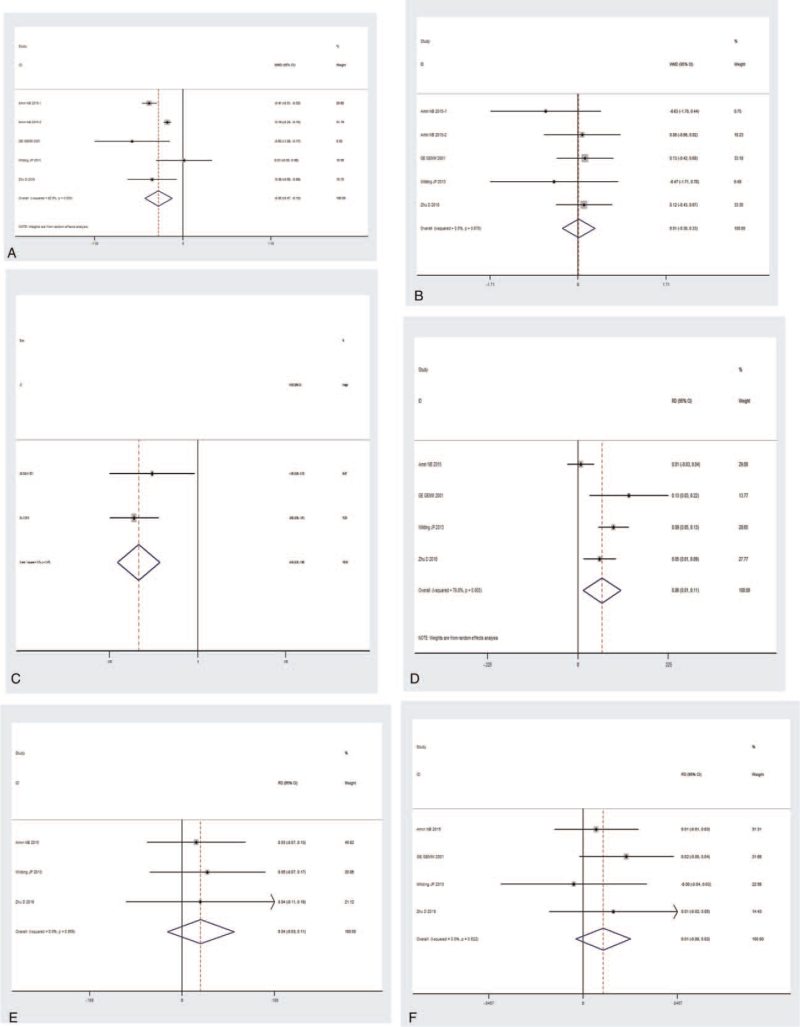
Outcomes of the comparison of the HbA_1_c (A). FBG (B). 2-h PPG (C). hypoglycemia (D). Adverse events (E) and severe adverse (F) of the GKAs with placebo in patients with T2DM. 2-h PPG = 2-hour postprandial plasma glucose, GKAs = glucokinase activators, HbA_1c_ = glycated hemoglobin, T2DM = type 2 diabetes mellitus.

**Figure 3:**
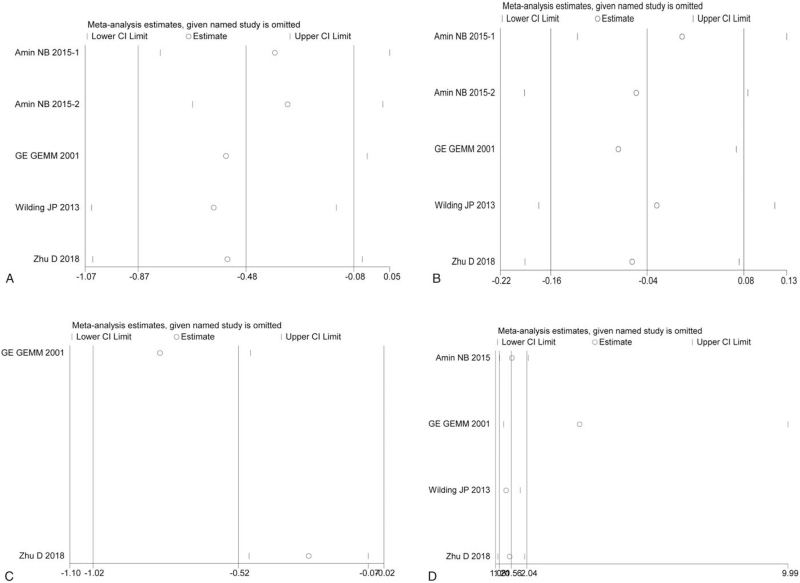
Outcomes of the comparison of the HbA_1_c (A). FBG (B). 2-h PPG (C), and hypoglycemia (D) of the GKAs with placebo in patients with T2DM according to sensitivity analysis. 2-h PPG = 2-hour postprandial plasma glucose, GKAs = glucokinase activators, HbA_1c_ = glycated hemoglobin, T2DM = type 2 diabetes mellitus.

All 5 studies reported a change in HbA_1c_ levels from baseline to the end of the study period. We carried out a random-effect meta-analysis that involved 1139 participants assigned to the GKAs group and 264 patients assigned to the placebo group. Analysis of the pooled data showed that GKAs were associated with significant reductions in HbA_1c_ levels from baseline (WMD, –0.3%; 95% CI, –0.466% to –0.134%) (Fig. 2A). Significant heterogeneity was present for studies reporting changes in HbA_1c_ (*I*^2^ = 82.9%, *P* = .000). To eliminate heterogeneity, we conducted subgroup analyses based on the types of GKAs (Table 3). However, heterogeneity was also present in the partial GKA group (*I*^2^ = 89.9%, *P* = .000). Sensitivity analyses showed that exclusion of any study did not significantly change the estimate (Fig. 3A). Egger and Begg tests for each of the 5 studies indicated no significant publication bias (*P* = .506 and *P* = .806, respectively).

**Table 3:** Subgroup analysis based on the types of GKAs.

			Heterogeneity		
	Comparisons	Patients	*P*	*I* ^2^	Estimated WMD (95% CI)
HbA1c					
Partial GKAs	3	667/149	0	89.90%	−0.242 (−0.436 to −0.048)
Full GKAs	2	674/168	.383	0.00%	−0.454 (−0.705 to −0.202)

			Heterogeneity		
	Comparisons	Patients	*P*	*I* ^2^	Estimated RD (95% CI)
Hypoglycemia					
Partial GKAs	2	677/205	.001	90.20%	0.047 (–0.033–0.127)
Full GKAs	2	677/168	.177	45.20%	0.076 (0.01–0.142)

CI = confidence interval, GKAs = glucokinase activators, HbA1c = glycated hemoglobin, RD = risk difference, WMD = weighted mean difference.

#### 3.3.2 FBG

All 5 studies reported changes in fasting plasma glucose (FPG) levels from baseline to the end of the study period. Fixed effects meta-analyses showed no significant differences between GKAs and placebos in fasting plasma glucose levels (WMD 0.013 mmol/L; 95% CI, −0.304 to 0.33 mmol/L) (Fig. 2B). There was no heterogeneity among all studies (*I*^2^ = 0%, *P* = .768). Sensitivity analyses showed that exclusion of any study did not significantly change the estimate (Fig. 3B).

#### 3.3.3 2-h PPG

Two studies reported changes in 2-h PPG levels from baseline to the end of the study period. Fixed effects meta-analyses showed a significantly greater change in 2-h PPG levels (WMD −2.434 mmol/L; 95% CI, −3.304 to −1.564 mmol/L) than that observed with the placebo groups (Fig. 2C). There was no heterogeneity among these 2 studies (*I*^2^ = 0%, *P* = .476). Sensitivity analyses showed that exclusion of any study did not significantly change the estimate (Fig. 3C).

### 3.4 Safety outcomes

#### 3.4.1 Hypoglycemia

Of the 4 studies that reported data on hypoglycemia, random-effects meta-analyses showed that GKAs were associated with a higher prevalence of hypoglycemic events than placebos (RD, 0.06; 95% CI 0.013–0.106) (Fig. 2D). Mild heterogeneity existed in all 4 studies (*I*^2^ = 76.8%, *P* = .005). To eliminate heterogeneity, we conducted subgroup analyses based on the types of GKAs (Table 3). According to these analyses, full GKAs were found to be associated with a higher prevalence of hypoglycemic events (full GKAs: RD, 0.076; 95% CI, 0.01–0.142), while partial GKAs had no effect on hypoglycemia (partial GKAs: RD, 0.047; 95% CI, –0.033–0.127). However, heterogeneity was also present in the partial GKAs group (*I*^2^ = 90.2%, *P* = .001). Sensitivity analyses showed that exclusion of any study did not significantly change the estimate (Fig. 3D).

#### 3.4.2 AEs

A fixed-effects meta-analysis showed that the prevalence of AEs was similar between GKAs and placebos (RD, 0.038; 95% CI, −0.03–0.106) (Fig. 2E). There was no heterogeneity among the selected 4 studies (*I*^2^ = 0%, *P* = .959).

#### 3.4.3 Serious adverse events (SAEs)

A fixed-effects meta-analysis showed that the prevalence of SAEs was similar between GKAs and placebos (RD, 0.01; 95% CI, −0.004–0.023) (Fig. 2F). There was no heterogeneity among the selected 4 studies (*I*^*2*^ = 0%, *P* = .622).

## 4 Discussion

Mounting evidence implicates β-cell dysfunction as the primary cause associated with the progression of T2DM, and defective early phase insulin release has been clearly demonstrated in patients with the disease.^[16]^ There are many antidiabetic agents with different mechanisms; however, a number of patients with diabetes have experienced frustrations from poor glycemic control despite adherence.^[17]^ There is an urgent need for clinically differentiated oral antidiabetic agents to address drivers of β-cell dysfunction and improve the function of the defective glucose sensor. GKAs are a relatively new therapeutic class of oral anti-hyperglycemic agents for managing T2DM. In order to provide new medical evidence for clinical treatment, it is necessary to study the efficacy and safety of GKAs in the management of this disease. In this meta-analysis of 5 double-blind RCTs, we compared the effects wrought by GKAs with those of placebos. Some conclusions on the efficacy and safety could be obtained from a pooled analysis of 1139 patients with T2DM. Compared with the placebo group, the GKA-treated group had a stronger effect on glucose control; however, these novel agents had an increased risk of developing hypoglycemia. Nonetheless, GKAs could be an attractive choice for the treatment of T2DM.

Glucokinase is mainly expressed in pancreatic β-cells and the liver, where it catalyses the phosphorylation of glucose to glucose-6-phosphate.^[18]^ In the liver, glucokinase processes glucose after a meal and converts it into glycogen in a glucose-dependent manner.^[1]^ Therefore, the enzyme functions as a glucose sensor in pancreatic β-cells. In normal physiological states, the blood glucose level is maintained between 3.9 and 5.6 mmol/L (homeostasis) to ensure that the brain, red blood cells, and other glucose dependent cells remain in a healthy state. This condition is called glucose homeostasis. As a glucose sensor, glucokinase can sensitively recognize the change in glucose concentration, timely regulate the secretion of insulin and glucagon, and maintain glucose homeostasis. Impaired function and expression of the enzyme has been observed in patients with T2DM.^[3–5]^ Activation of glucokinase enhances glucose phosphorylation, increasing glucose-stimulated insulin secretion, and hepatic glucose uptake, as well as decreasing hepatic glucose output^[19]^^.^ Therefore, this enzyme has been proposed as a therapeutic target as it offers the potential for dual effects in correcting 2 underlying defects in T2DM, viz. impaired insulin secretion and excessive hepatic glucose output.^[20]^

To date, 10 GKAs (PF-04937319,^[6]^ MK-0941,^[7]^ AZD1656,^[8]^ dorzagliatin [HMS5532],^[10]^ PF-04991532,^[21]^ piragliatin [RO4389620],^[22]^ AMG 151 [ARRY-403],^[23]^ AZD6370,^[24]^ globalagliatin [LY2608204],^[25]^ and TTP399^[26]^) have undergone clinical trial investigations; however, the results from these studies were not consistent. Due to the obvious heterogeneity among the studies, we developed strict inclusion criteria for a meta-analysis. Many studies were excluded because they did not meet the inclusion criteria.^[22–24]^ In addition, the study involving TTP399, a hepatoselective GKA, was excluded because the results could not be extracted.^[26]^ The GKAs included in the meta-analysis were PF-04937319 (N = 2), MK-0941 (N = 1), AZD1656 (N = 1), and dorzagliatin (N = 1). Our meta-analysis showed that GKAs were associated with significant reductions in HbA_1c_ levels from baseline (WMD, −0.3%; 95% CI, −0.466% to −0.134%). In terms of function, GKAs can be divided into 3 categories: liver selective GKAs which can only activate glucokinase of the liver, partial GKAs which act on the liver and pancreas at the same time and reduce the Km value of glucokinase, and full GKAs which act on both the liver and pancreas and can increase the Vmax of glucokinase and decrease its Km. PF-04937319 and AZD1656 are partial activators of glucokinase, while MK-0941 and dorzagliatin are all full GKAs. According to the subgroup analyses involving different types of GKAs, both partial and full GKAs were seen to reduce HbA_1c_ levels (partial GKAs: WMD, −0.242%; 95% CI, −0.436% to −0.048%) (full GKAs: WMD, −0.454%; 95% CI, −0.705% to −0.202%). Our meta-analysis showed that GKAs induced a significantly greater change in 2-h PPG levels (WMD −2.434 mmol/L; 95% CI, −3.304 to −1.564 mmol/L) but had no effect on fasting blood glucose (WMD 0.013 mmol/L; 95% CI, −0.304 to 0.33 mmol/L) than the placebo. The observed reduction in HbA_1c_ appeared to be the result of changes in postprandial blood glucose levels.

With regard to safety and tolerability, our meta-analysis showed that GKAs carried a higher risk of hypoglycemic events than placebos. According to the subgroup analyses, we found that the main cause of hypoglycemia were full GKAs. The increase in the incidence of hypoglycemia is related to the degree of activating the glucokinase enzyme which is consistent with triggering mutations of this protein that are associated with varying degrees of hypoglycemia.^[27]^ The risk of SEs and SAEs were relatively low. Overall, GKAs were well tolerated.

Our study had strengths (e.g., it was conducted using a systematic approach); however, some limitations are noted. First, most of the studies included in our meta-analysis were of a relatively short duration (longest duration was 30 weeks). Therefore, we could not comment on long-term efficacy and safety. Second, despite the addition of more RCTs, statistical heterogeneity was detected. This heterogeneity may have arisen from the differences in study design, duration of T2DM, and control drug therapy. Third, the number of included studies was relatively small. Fourth, the cut-off of hypoglycemia was not clearly defined in the included studies. Therefore, more “high-quality” RCTs are needed to verify our conclusions.

## 5 Conclusions

Among patients with T2DM, GKAs were more effective than placebo treatment for postprandial blood glucose control. In addition, GKAs had a comparably high risk of causing hypoglycemia. However, this new class of hypoglycemic agents needs continued evaluation in RCTs to ascertain long-term efficacy and safety.

## Author contributions

**Conceptualization:** Qian Gao.

**Data curation:** Qian Gao, Wenjun Zhang.

**Investigation:** Tingting Li.

**Methodology:** Tingting Li.

**Resources:** Wei Zhu.

**Software:** Naijun Chen.

**Supervision:** Wenjun Zhang, Guojun Yang, Huawei Jin.

**Writing – original draft:** Qian Gao.

**Writing – review & editing:** Qian Gao.
